# The mediating effects of self-directed learning and nursing professional values on the relationship between digital literacy and narrative competence among nursing students: a cross-sectional study

**DOI:** 10.1186/s12909-026-08757-z

**Published:** 2026-02-12

**Authors:** Ya Meng, Xin Jia, Tingting Hou, Yufang Deng, Jian Song

**Affiliations:** 1 Department of Nursing, Zhengzhou Health Vocational College, Zhengzhou, China; 2 Office of Academic Affairs, Zhengzhou Health Vocational College, Zhengzhou, China; 3 Department of Nursing, Jingzhou Institute of Technology, Jingzhou, China

**Keywords:** Narrative competence, Digital literacy, Self-directed learning, Nursing professional values, Structural equation modeling

## Abstract

**Objective:**

This study aimed to explore the degree of narrative competence and determine how it was predicted by digital literacy, self-directed learning and nursing professional values among nursing students in China using a structural equation modeling (SEM)-based approach.

**Methods:**

A cross-sectional survey was conducted among 1,025 nursing students identified through convenience cluster sampling. Data were collected on December 2024 via the Wenjuanxing online questionnaire platform. The data were analyzed using descriptive statistics and SEM.

**Results:**

The mean narrative competence score of the nursing students was 146.71 ± 17.94. Digital literacy (*β* = 0.20, *P* < 0.001), self-directed learning (*β* = 0.43, *P* < 0.001), and nursing professional values (*β* = 0.27, *P* < 0.001) were positive predictors of narrative competence. Among these variables, self-directed learning had the most significant influence on the degree of narrative competence, and nursing professional values mediated the relationship between digital literacy and narrative competence.

**Conclusions:**

Nursing students’ digital literacy both directly and indirectly (via self-directed learning and nursing professional values) influences their narrative competence. Improving nursing students’ digital literacy and self-directed learning skills can improve humanistic care, nursing quality and nurse‒patient relationships.

## Background

With the implementation of the Healthy China strategy and the transformation of healthcare service models, nursing is shifting from technology dominance to human-technology synergy. This approach resonates profoundly with the narrative medicine principle, which involves listening to patients’ stories, thereby highlighting the significance of narrative competence in modern nursing practice.

Narrative competence, a critical dimension of humanistic medicine, was defined by Charon [1] as a systematic capacity to acknowledge, absorb, interpret, and respond to patients’ stories and dilemmas, fundamentally enabling empathic connections and therapeutic interventions through listening, understanding, and responding.

Compared with traditional nursing communication, narrative care integrates three core competencies: narrative sensitivity (capturing verbal/nonverbal cues), narrative reflexivity (reconstructing stories to identify unmet needs), and narrative agency (translating insights into personalized care) [2]. This competency framework is not only essential for good communication between medical staff and patients but also a powerful instrument for delivering holistic healthcare [3, 4].

Research into narrative competence among nurses has demonstrated significant benefits worldwide. Studies have indicated that it enhances nurses’ clinical reasoning, observational skills, empathy, resilience, and therapeutic relationships [5–7]. Educational interventions, such as reflective writing, close reading of literature, and narrative pedagogy, have been developed and shown to be effective at cultivating these skills among nursing students and practitioners [8].

In China, recent studies have focused on developing educational programs for nursing students and exploring their effects on empathy and communication skills [9, 10]. Additionally, existing research lacks critical comparisons of findings, and key gaps remain: few studies have quantified the measurement properties of narrative competence or systematically examined its outcomes, particularly in the context of digital healthcare transformation.

In the era of intelligent healthcare, the cultivation of digital literacy and narrative competence holds equal significance. As emphasized in the Digital Health White Paper 2023 [11], next-generation nursing professionals must integrate technological acuity with narrative empathy. Digital literacy, defined as the awareness, attitude, and ability to appropriately identify, access, manage, integrate, evaluate, and synthesize digital resources using technological tools [12], has permeated every facet of nursing practice. From electronic health record (EHR) management systems to intelligent patient monitoring devices and online pharmaceutical databases and mobile nursing terminals, digital technologies have permeated the entire chain of nursing work [13, 14]. Accordingly, digital literacy is considered an important competency among healthcare providers in healthcare systems [15, 16].

Although nursing students generally hold positive attitudes toward digital technologies and are frequently exposed to them in daily life, they still demonstrate insufficient confidence in the use of discipline-specific digital tools and software [14, 17]. Systematically improving their digital literacy is thus critical for advancing national health informatization strategies and adapting to medical service digitalization, requiring integration of targeted interventions into nursing curricula [18], including curricula related to humanities education and communication training, which are closely linked to the development of narrative competence.

Nursing professional values are defined as the core beliefs and principles, including altruism, human dignity, integrity, and social justice, that guide nurses’ decision-making and patient advocacy [19]. Extensive research has confirmed the role of nursing professional values in improving clinical decision-making, ethical sensitivity, and patient satisfaction [20]. Self-directed learning (autonomously diagnosing needs, setting goals, and evaluating outcomes [21]) is essential for nurses to adapt to evolving healthcare technologies [22].

Existing studies have highlighted the close association between professional values and caring behaviors in specific nursing contexts, such as intensive care units and neonatal intensive care units [23, 24]. As a crucial component of caring behavior, narrative competence is essential for improving nurse-patient relationships and nursing quality. However, no study has quantified how nursing professional values and self-directed learning interact with narrative competence—especially in the context of digital literacy cultivation for Chinese nursing students, which is a critical research gap that constitutes the core novelty of the present study.

To address the identified research gap, the following refined research questions are proposed: 1. What is the level of narrative competence among Chinese nursing students? 2. What are the direct and indirect predictive effects of digital literacy, self-directed learning, and professional values on nursing students’ narrative competence, as analyzed via structural equation modeling (SEM)?

**Fig. 1:**
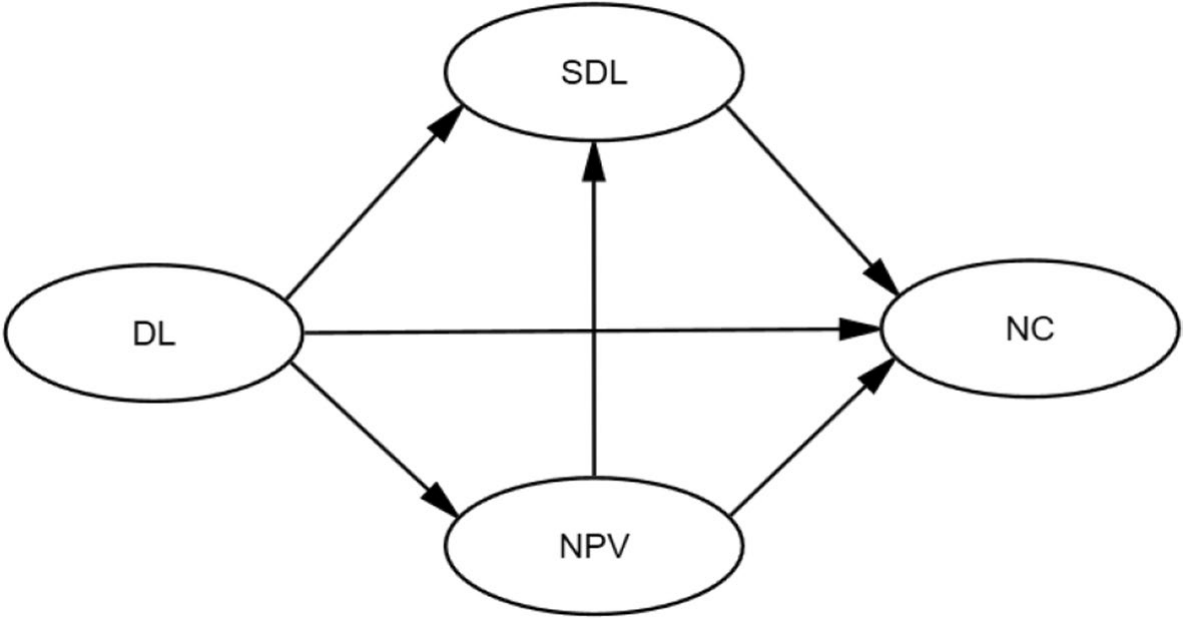
Conceptual framework of the study

To further clarify the theoretical rationale underlying the above hypotheses and the intrinsic links among variables, a detailed conceptual framework is presented below (Fig. 1).

Guided by these research questions and the conceptual framework, we constructed an SEM hypothesis model with three specific hypotheses: (1) Digital literacy (DL) has a direct positive predictive effect on narrative competence (NC) (DL → NC); (2) Digital literacy (DL) has an indirect positive predictive effect on narrative competence (NC) through self-directed learning (SDL) (DL → SDL → NC); (3) Digital literacy (DL) has an indirect positive predictive effect on narrative competence (NC) through nursing professional values (NPV) (DL → NPV → NC).

## Methods

### Study design and participants

This study adopted a convenience cluster sampling method, and the clusters were selected on the basis of the accessibility and willingness of nursing colleges to cooperate. The research setting was two medical colleges in Henan Province, China, and 20 classes (clusters) were selected from each college. The inclusion and exclusion criteria were applied at the individual level: inclusion criteria were (1) full-time nursing students and (2) voluntary participation in this study; exclusion criteria were (1) students who were not majoring in nursing and (2) students who were on leave or dropped out during the data collection period.

In accordance with the rule-of-thumb for SEM sample size (the sample size should be at least 10 times the number of observed variables and, ideally, 20 times), this study included 4 latent variables and 21 observed variables. Therefore, the minimum required sample size is 210, and the actual sample size of 1025 in this study is far greater than the minimum requirement, which ensures the reliability and validity of the SEM analysis.

From the initial pool of 1,132 completed questionnaires, 107 submissions (9.46%) were excluded during systematic data screening. The exclusion protocol eliminated responses demonstrating patterned answering behavior (e.g., straight-line responding) and those with excessively short response durations (< 240 s). Consequently, 1,025 valid questionnaires were retained for the final analysis, for a 90.55% valid response rate.

### Instruments

#### Sociodemographic characteristics

The sociodemographic variables included key demographic characteristics, such as age, gender, academic year, residence, family environment (i.e., a family interaction mode characterized by equal communication between family members, respect for individual opinions, and joint participation in family decision-making), self-directed course of study upon admission, such as the nursing major, and practicum experience.

#### Narrative Competence Scale, NCS

The Narrative Competence Scale (NCS), developed by Ma WZ [25] in 2020, is an original Chinese instrument for assessing medical narrative competence among healthcare practitioners. This tool consists of 27 items distributed across three dimensions: listening (9 items), understanding (12 items), and reflecting (6 items). The responses range from 1 (completely disagree) to 7 (completely agree) on a 7-point Likert scale, yielding a total score that ranges from 27–189, with higher scores indicating higher narrative competence. The score can be interpreted according to the following thresholds: scores below 145 indicate weak competence, 145–163 represent moderate competence, and scores exceeding 163 indicate strong competence. Cronbach’s α coefficient of the scale is 0.950, and the test–retest reliability is 0.717 [25]. In this sample, Cronbach’s α value of the scale was 0.948.

#### College students’ digital literacy scale

The College Students’ Digital Literacy Scale was developed by Sheng SY [24] in 2022. This 20-item scale, an original Chinese version, consists of six dimensions: tools and technology (4 items), information and data (3 items), communication and collaboration (3 items), digital content creation (3 items), problem-solving (3 items), and security (4 items). The responses range from 1 (completely disagree) to 5 (completely agree) on a 5-point Likert scale, yielding total scores ranging from 20 to 100. Reliability analysis revealed a Cronbach’s α coefficient of 0.941, and the KMO value of 0.929 demonstrates that the questionnaire has good validity [26]. In this study, the scale achieved a Cronbach’s α coefficient of 0.918.

#### Nursing Professional Values Scale, NPVS

The Nursing Professional Values Scale (NPVS), originally developed by Weis [19] and cross-culturally adapted by Chen et al. [27], is designed to evaluate professional values among nurses and nursing students. This 26-item scale consists of four dimensions: caring (10 items), activism (8 items), professionalism (5 items), and trust (3 items). Items are rated on a 5-point Likert scale, with a score ranging from not important (1) to most important (5), yielding total scores ranging from 26 to 130. Higher total scores reflect stronger ratings and orientation for professional values. The scale demonstrates established reliability and validity, with original subscale Cronbach’s α coefficients between 0.73 and 0.87 and a test–retest reliability of 0.88 [27]. In the current study, the overall Cronbach’s α coefficient was 0.955.

#### Self-Directed learning readiness scale

This study utilized the Self-Directed Learning Readiness Scale, which was originally developed by Fisher et al. [28] and subsequently adapted into Chinese by Zhang et al. [29], to evaluate nursing students’ autonomous learning competence. This 30-item instrument comprises four distinct dimensions: learning motivation (8 items), self-management skills (11 items), cooperative learning skills (5 items) and information literacy (6 items). All the items employ a 5-point Likert scale, with scores ranging from 1 (not at all) to 5 (exactly). The total scores range between 30 and 150, with higher scores indicating greater self-directed learning capacity. The internal consistency reliability of the scale is 0.822 [29]. In this sample, the Cronbach’s alpha value of the scale was 0.914.

### Data collection

Data were collected through a web-based survey platform (Wenjuanxing, www.wjx.cn) using anonymous self-administered questionnaires. To prevent duplicate submissions, we set device restrictions on the Wenjuanxing online questionnaire platform. With institutional approval from the nursing faculty, a standardized instruction session was conducted prior to survey administration to ensure participants’ comprehensive understanding of the study objectives, ethical considerations, and technical requirements.

Nursing students voluntarily accessed the questionnaire by scanning a designated QR code and undertook the questionnaire on an individual basis, without any external assistance. The first page of the questionnaire presented a detailed informed consent form, including the study’s purpose, content, risks, benefits, and participants’ right to withdraw at any time without prejudice. Participants could only proceed to the formal questionnaire by clicking “Agree and Continue”; clicking “Disagree” terminated the questionnaire automatically. The researcher subsequently retrieved the collected data via the platform’s backend system. To improve data quality control, mandatory response settings were implemented for all the items, coupled with a minimum completion time threshold of 240 s as determined through pilot testing, effectively preventing random or insincere responses from being collected for the study.

### Statistical analysis

The Statistical Package for the Social Sciences (SPSS) version 25.0 (IBM, New York, USA) was used for data analyses. The normality of continuous variables was tested by analyzing skewness and kurtosis values. Continuous variables with a normal distribution are expressed as the mean ± standard deviation (SD). Comparisons between groups were performed by independent-samples t tests or one-way ANOVA. Pearson’s correlation analysis was used to assess the associations between the main variables. Additionally, the variance inflation factor (VIF) was calculated to test for multicollinearity (VIF < 10 indicates no serious multicollinearity). AMOS software version 24.0 was used to define the direct, indirect and total effects of different variables on narrative competence. The standardized regression coefficient (*β*) in the path model was used to understand the effect of an independent variable on a dependent variable.

To test for mediation effect testing, the bootstrap method with 5,000 resamples was adopted, and the 95% confidence interval (CI) was used to judge the significance of indirect effects. The goodness-of-fit of the model was confirmed using the CMIN/df (< 5) (the large sample size inflates the χ^2^ value, leading to a higher CMIN/df), root mean square error of approximation (RMSEA < 0.08), goodness-of-fit index (GFI > 0.95), and comparative fit index (CFI ≥ 0.90) [30]. For all the data analyses, the test level α was set to 0.05.

## Results

### Participant characteristics

The mean age of the 1025 participants was 18.72 (SD = 0.90) years. The sample comprised 13.3% male and 86.7% female participants. With respect to family characteristics, 8.6% were only children, while 91.4% had siblings. In terms of geographic origin, 25.3% came from urban areas, 24.2% from suburban regions, and 50.5% from rural localities. In terms of academic year, 74.1% were freshmen and 25.9% were sophomores. With respect to practicum experience, 10.7% had practicum experience, while 89.3% had no practicum experience.

### Comparison of narrative competence among nursing students with different demographic characteristics

This study revealed significant differences in narrative competence among nursing students on the basis of their demographic and psychosocial factors. Students with leadership experience (*t* = 2.963, *P* = 0.003) and who experienced democratic family dynamics (*F* = 16.958, *P* < 0.001) had significantly greater narrative competence scores. Similarly, liking the nursing major was also associated with greater narrative competence. Personality traits exhibited a dose‒response relationship: very extroverted individuals scored 18.84 points higher than their introverted peers did (*F* = 11.345, *P* < 0.001). In contrast, gender, residence, and practicum experience were not significantly related to narrative competence (*P* > 0.05). Further details can be found in Table 1.

**Table 1:** Comparison of narrative competence among nursing students with different demographic characteristics (Mean ± SD)

Variables	n	%	Total score of narrative competence	*t*/*F*	*P*
Gender					
Male	136	13.3	145.76 ± 17.63	−0.663	0.508
Female	889	86.7	146.85 ± 18.00		
Residence					
Urban	259	25.3	147.51 ± 18.45		
Rural	518	50.5	145.85 ± 17.88	1.214	0.279
Village	248	24.2	147.67 ± 17.51		
Student leader					
Yes	320	31.2	149.16 ± 17.47	2.963	0.003
No	705	68.8	145.59 ± 18.06		
Academic year					
Freshman	760	74.1	147.94 ± 17.65	3.743	< 0.001
Sophomore	265	25.9	143.18 ± 18.33		
Family atmosphere					
Highly democratic	275	26.8	151.84 ± 18.39		
Moderately democratic	588	57.4	145.32 ± 17.24	16.958	< 0.001
Less democratic	162	15.8	143.02 ± 17.97		
Character					
Very extroverted	34	3.3	157.44 ± 15.29		
More extroverted	211	20.6	149.93 ± 16.66		
Neutral	505	49.3	147.47 ± 18.34	11.345	< 0.001
More introverted	250	24.4	141.81 ± 17.83		
Very introverted	25	2.4	138.60 ± 16.46		
Voluntary selection upon admission					
Be transferred	94	9.2	142.97 ± 18.01		
Personal interest	158	15.4	154.57 ± 17.43	14.597	< 0.001
Family/friends influence	458	44.7	144.42 ± 17.94		
Easy to find employment	315	30.7	147.21 ± 17.04		
Like the nursing major					
Yes	675	65.9	149.51 ± 17.49	7.102	< 0.001
No	350	34.1	141.31 ± 17.60		
Practicum experience					
Yes	110	10.7	148.53 ± 18.49	1.126	0.260
No	915	89.3	146.49 ± 17.87		

Post hoc comparisons using the LSD method were further conducted to identify pairwise differences between groups. Briefly, participants from families with a highly democratic atmosphere achieved significantly higher narrative competence scores than did those from families with a moderately democratic or less democratic atmosphere (all *ps* < 0.01). Among the different character types, highly extroverted participants scored significantly higher than those in all the other character subgroups did (all *ps* < 0.05). With respect to program choice, students who voluntarily selected the nursing program obtained higher narrative competence scores than their counterparts in other program choice groups did (all *ps* < 0.01).

### Descriptive statistics

The measured scores (mean ± SD) of the various instruments for the nursing students were as follows: narrative competence (146.71 ± 17.94), digital literacy (64.79 ± 10.70), nursing professional values (114.78 ± 11.23), and self-directed learning (104.37 ± 14.17). For narrative competence, in particular, scores were further categorized into three levels according to the predefined thresholds: weak (*n* = 459, 44.8%), moderate (*n* = 401, 39.1%), and strong (*n* = 165, 16.1%). A detailed breakdown of these scores across all dimensions is presented in Table 2.

**Table 2:** Descriptive statistics for the variables (*n* = 1025)

Dimensions	Items	Observed scoring range	Average score
Narrative competence	27	65–189	146.71 ± 17.94
Listening	9	24–63	48.92 ± 5.99
Understanding	12	24–84	64.82 ± 8.89
Reflecting	6	15–42	32.97 ± 4.40
Digital literacy	20	24–100	64.79 ± 10.70
Tools and technology	4	4–20	11.30 ± 2.76
Information and data	3	3–15	9.44 ± 2.10
Communication and collaboration	3	3–15	9.91 ± 2.03
Digital content creation	3	3–15	9.92 ± 1.97
Problem-solving	3	3–15	9.34 ± 2.15
Security	4	4–20	14.87 ± 2.92
Nursing professional values	26	68–130	114.78 ± 11.23
Caring	10	25–50	44.97 ± 4.42
Activism	8	21–40	33.78 ± 4.04
Professionalism	5	11–25	22.25 ± 2.30
Trust	3	8–15	13.78 ± 1.38
Self-directed learning	30	45–144	104.37 ± 14.17
Learning motivation	8	12–40	28.47 ± 4.48
Self-management skills	11	13–55	38.07 ± 6.29
Cooperative learning skills	5	9–25	16.99 ± 2.65
Information literacy	6	6–30	20.85 ± 3.36

### Bivariate correlation of different variables

As shown in Table 3, Pearson correlation analysis revealed significant positive correlations between nursing students’ narrative competence (including its dimensions) and the total scores as well as individual dimensions of digital literacy, nursing professional values, and self-directed learning (*P* < 0.01). Notably, multiple pairwise correlation tests were conducted in this study, which may increase the risk of Type I error.

**Table 3:** Correlation analysis among different variables

	**Narrative competence**	Listening	Understanding	Reflecting
Digital literacy	0.460^**^	0.413^**^	0.432^**^	0.441^**^
Tools and technology	0.216^**^	0.187^**^	0.205^**^	0.214^**^
Information and data	0.341^**^	0.315^**^	0.318^**^	0.322^**^
Communication and collaboration	0.350^**^	0.314^**^	0.336^**^	0.319^**^
Digital content creation	0.376^**^	0.345^**^	0.350^**^	0.356^**^
Problem-solving	0.374^**^	0.303^**^	0.364^**^	0.377^**^
Security	0.463^**^	0.436^**^	0.423^**^	0.441^**^
Nursing professional values	0.504^**^	0.456^**^	0.471^**^	0.482^**^
Caring	0.461^**^	0.429^**^	0.426^**^	0.437^**^
Activism	0.499^**^	0.434^**^	0.477^**^	0.480^**^
Professionalism	0.463^**^	0.422^**^	0.430^**^	0.445^**^
Trust	0.389^**^	0.365^**^	0.353^**^	0.375^**^
Self-directed learning	0.603^**^	0.520^**^	0.582^**^	0.577^**^
Learning motivation	0.509^**^	0.411^**^	0.502^**^	0.502^**^
Self-management skills	0.549^**^	0.453^**^	0.538^**^	0.535^**^
Cooperative learning skills	0.369^**^	0.423^**^	0.306^**^	0.311^**^
Information literacy	0.547^**^	0.462^**^	0.538^**^	0.513^**^

Note: ** represents *P* < 0.01

Additionally, a multicollinearity test was performed by calculating the variance inflation factor (VIF) for the key predictor variables and the outcome variable. The results revealed that the VIF values of all the variables were less than 2 (NC: 1.901; DL: 1.339; SDL: 1.711; NPV: 1.384).

### Structural equation model

On the basis of preliminary correlation analyses, a theoretical model was developed to examine the determinants of nursing students’ narrative competence. In this conceptual framework, narrative competence was used as an endogenous outcome variable, digital literacy was used as an exogenous latent variable, and self-directed learning and nursing professional values were used as dual mediating constructs. Structural equation modeling was subsequently performed to empirically validate and refine the hypothesized relationships.

Using maximum likelihood (ML) estimation with iterative adjustments, the final model demonstrated acceptable fit indices (absolute fit: χ^2^/df = 5.610; GFI = 0.935; AGFI = 0.904; RMSEA = 0.067; incremental fit: NFI = 0.957; IFI = 0.964; TLI = 0.952; CFI = 0.964). All indices except χ^2^/df were within the ideal range; considering the large sample size effect (1,025 valid samples), the χ^2^/df value was acceptable.

**Fig. 2:**
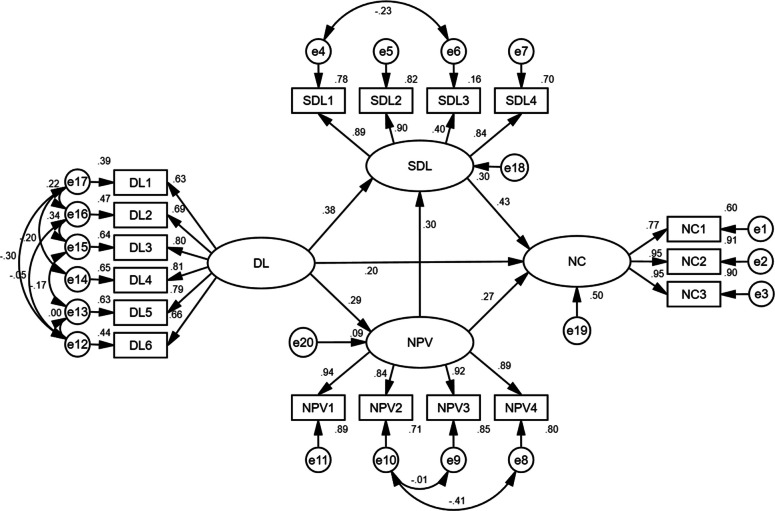
Standardized regression weights for the modified structural equation modeling

Standardized path coefficient analysis (Fig. 2) revealed significant predictive relationships: digital literacy was directly positively associated with narrative competence (*β* = 0.201, *P* < 0.001), whereas self-directed learning aptitude was the strongest predictor (*β* = 0.428, *P* < 0.001), and nursing professional values demonstrated moderate mediating effects (*β* = 0.266, *P* < 0.001).

Table 4 presents the results of the structural equation model analysis. All three predictors demonstrated statistically significant positive associations with narrative competence (*P* < 0.001 for all), with self-directed learning having the strongest effect (*β* = 0.428, CR = 12.861), followed by nursing professional values (*β* = 0.266, CR = 9.428) and digital literacy (*β* = 0.201, CR = 6.618). The model for self-directed learning indicated substantial impacts from both digital literacy (*β* = 0.384, CR = 10.929) and nursing professional values (*β* = 0.296, CR = 9.468), with all paths significant at *P* < 0.001. Notably, digital literacy also significantly predicted nursing professional values (*β* = 0.292, *P* < 0.001).

**Table 4:** Parameter estimates of the modified model of narrative competence among nursing students (*n* = 1025)

Dependent variable	Independent variable	Parameter estimate	Standardized estimate	SE	C.R	*P*
Narrative competence	Digital literacy	0.484	0.201	0.073	6.618	< 0.001
	Nursing professional values	0.998	0.266	0.106	9.428	< 0.001
	Self-directed learning	0.500	0.428	0.039	12.861	< 0.001
Self-directed learning	Digital literacy	0.790	0.384	0.072	10.929	< 0.001
	Nursing professional values	0.950	0.296	0.100	9.468	< 0.001
Nursing professional values	Digital literacy	0.187	0.292	0.022	8.513	< 0.001

The standardized direct, indirect, and total effects of narrative competence are systematically presented in Table 5. Nursing professional values showed a comparable yet slightly stronger standardized effect size (*β* = 0.998, *P* < 0.001), suggesting the critical role of this variable in mediating narrative competence.

**Table 5:** Standardized direct, indirect and total effects of the modified model

Dependent variable	Independent variable	Direct effect	Indirect effect	Total effect
Narrative competence	Digital Literacy	0.484(0.305–0.645)	0.670(0.562–0.802)	1.153(0.983–1.323)
	Nursing Professional Values	0.998(0.818–1.345)	0.475(0.358–0.628)	1.472(1.257–1.788)
	Self-directed learning	0.500(0.419–0.611)		0.500(0.419–0.611)

## Discussion

This study aimed to explore the status of narrative competence among nursing students and clarify the associations among digital literacy, nursing professional values, self-directed learning, and narrative competence using structural equation modeling (SEM). The key findings can be summarized as follows: (1) the overall narrative competence of nursing students was moderate; (2) self-directed learning showed the strongest positive association with narrative competence, followed by professional values and digital literacy; and (3) digital literacy exerted both direct and indirect effects on narrative competence through self-directed learning and professional values.

To address the first research aim of exploring the status of nursing students’ narrative competence, the study revealed nursing students’ narrative competence scores (146.71 ± 17.94 points overall), indicating moderate competence, which were slightly lower than the findings of Ma et al. [25]. According to the Narrative Competence Scale (NCS) criteria, in which scores below 145 indicate weak competence and scores above 163 indicate strong competence, only 16.1% of the nursing students in our group demonstrated strong narrative competence. This moderate level of competence is consistent with the characteristics of nursing students in the pre-clinical stage, who have limited opportunities to engage in real patient narrative interactions. A randomized controlled trial focusing on narrative medicine education confirmed that systematic narrative training based on the ADDIE model could effectively enhance nursing students’ narrative competence and empathy levels [31]. In contrast, the nursing students in our study lack such standardized narrative medicine curricula, which may be a key reason for their relatively lower narrative competence scores. This relatively low competence level may stem from two factors: (1) narrative medicine remains a relatively novel concept for nursing students [10], and (2) despite the trainable nature of narrative competence, there is a lack of sufficient narrative care training programs in current nursing curricula [31].

Among the three dimensions of narrative competence, the reflecting dimension scored the highest, while the understanding dimension scored the lowest, suggesting that nursing students can actively develop care strategies through patient narratives but struggle to accurately identify patients’ narrative needs. These findings highlight the need for nursing educators to prioritize understanding cultivation through systematic training, fostering students’ ability to effectively listen, interpret, reflectively write and reconstruct patients’ narratives while developing empathic reflection skills, ultimately realizing the therapeutic value of narrative medicine [10, 32, 33]. This is expected to be associated with improved nurse–patient relationships, reduced conflicts, and a supportive, positive healthcare environment, all of which contribute to the overall development of the healthcare industry and the enhancement of human health [34, 35].

To address the research aim of clarifying the associations among digital literacy, nursing professional values, self-directed learning, and narrative competence, the results from the structural equation model offer a more nuanced and comprehensive understanding of the associations among digital literacy, nursing professional values, self-directed learning, and narrative competence among nursing students. The significant standardized path coefficients not only validate the positive correlations identified earlier but also elucidate the directional and relative strength of these associations, though cross-sectional data preclude causal inferences.

Self-directed learning aptitude emerges as the factor with the strongest associative link to narrative competence (*β* = 0.428, *P* < 0.001), underscoring its associative role in the observed relationship with students’ narrative ability. These findings suggest that students who possess strong self-directed learning skills are more likely to actively seek out opportunities to enhance their narrative competence, engage in reflective practice, and continuously refine their communication techniques. Self-directed learners may independently explore resources [22], such as narrative-based educational materials or patient storytelling workshops, which are associated with the development of narrative skills [36]. Moreover, their ability to set goals, monitor progress, adapt learning strategies and engage in life-long learning [37] is linked to more effective internalization of narrative techniques, which in turn shows a strong association with their overall narrative competence.

Digital literacy also demonstrates a significant direct positive association with narrative competence (*β* = 0.201, *P* < 0.001). In the digital age of healthcare, proficiency in digital tools provides nursing students with expanded channels for narrative communication [38]. This finding aligns with the conclusions of a recent systematic review [39], which emphasized that the convergence of digital and narrative medicine constructs a compelling pedagogical framework for healthcare education. Enhancing awareness of current digital disparities and providing targeted support for digital skill development can equip students to become resilient, evidence-based practitioners capable of thriving in a dynamically evolving digital healthcare environment [40, 41]. The use of electronic health records, telemedicine platforms, and social media for patient engagement allows students to practice storytelling in diverse contexts, which in turn is associated with enhanced narrative competence [14, 42]. Additionally, digital literacy enables access to a vast array of multimedia narrative resources [43], such as video-recorded patient testimonials and virtual reality simulations of patient interactions, further enriching students’ learning experiences [41] and showing an associative link to the development of their narrative skills.​

Nursing professional values, with a parameter estimate of 0.998 and a standardized path coefficient of 0.266, show a crucial associative relationship with narrative competence. Consistent with humanistic nursing theory’s emphasis on the core position of humanistic care and patient-centered communication, the findings indicate that when students internalize values such as compassion, empathy, and patient advocacy, they are more inclined to adopt a narrative-centered mindset in patient interactions [44].

These findings suggest that nursing education should emphasize self-directed learning, integrate digital literacy training to enhance narrative skills, and reinforce professional values to foster narrative competence. Specifically, we recommend integrating curriculum modules on narrative medicine, simulation-based patient interaction training, and combined reflective writing with digital storytelling activities into nursing education to effectively foster students’ narrative competence.

This study has both theoretical and practical implications. Theoretically, it validates a predictive model of nursing students’ narrative competence in the Chinese context, enriching the global framework of narrative medicine in non-Western nursing education settings. Methodologically, it uses SEM to systematically explore the multi-path association mechanism between variables, providing a reference for subsequent related research on mechanism exploration. Practically, against the background of the national requirement to strengthen the development of humanistic nursing, the findings provide empirical evidence for targeted curriculum optimization in Chinese nursing education, guiding educators to balance technological literacy and training in humanistic care. Building on these implications, future research should develop interventions based on these observed associations and examine their practical impacts on patient care.

### Limitations

This study has several limitations that should be acknowledged.

First, the cross-sectional design employed in this research prevents the establishment of causal relationships between the variables, and specifically, it is unable to establish temporal precedence for the hypothesized mediating effects—a critical limitation, as mediation inherently implies a sequential order of variables. Longitudinal studies are therefore needed to confirm the directional, causal, and temporal sequence of these relationships.

Second, the sample was recruited using convenient cluster sampling, which may introduce selection bias and limit the generalizability of the results. Additionally, the sample has limited regional representativeness, further restricting the extrapolation of the findings to nursing students in other geographic areas. Future studies should use more diverse, geographically representative samples to enhance the external validity of the findings.

Third, the data were collected solely through self-report questionnaires. Self-report measures are susceptible to response biases, such as social desirability bias; more importantly, this single-source data collection approach may lead to potential same-source bias, where the correlations between variables could be artificially inflated because of common method variance. Both types of biases may have influenced the accuracy of the data. The incorporation of objective assessment tools, observational methods, or multisource data collection (e.g., teacher evaluations, clinical supervisor feedback) to evaluate narrative competence and other variables could provide more robust and reliable results.

## Conclusions

Digital literacy, self-directed learning, and nursing professional values all show significant positive associations with narrative competence among Chinese nursing students. Furthermore, self-directed learning and nursing professional values mediate the association between digital literacy and narrative competence, indicating that digital literacy is associated with improved narrative competence not only directly but also indirectly through its associations with improved self-directed learning and strengthened nursing professional values. Therefore, nursing education should adopt an integrated approach that simultaneously develops digital literacy, self-directed learning, and nursing professional values. Strengthening these competencies through targeted educational strategies may effectively improve narrative competence, thereby being associated with enhanced humanistic care, nursing quality, and nurse–patient relationships.

## Data Availability

Data is provided within the manuscript or supplementary information files.

## Abbreviations

DL: Digital literacy
DL1: Tools and technology
DL2: Information and data
DL3: Communication and collaboration
DL4: Digital content creation
DL5: Problem-solving
DL6: Security
NPV: Nursing professional values
NPV1: Caring
NPV2: Activism
NPV3: Professionalism
NPV4: Trust
SDL: Self-directed learning
SDL1: Learning motivation
SDL2: Self-management skills
SDL3: Cooperative learning skills
SDL4: Information literacy
NC: Narrative competence
NC1: Listening
NC2: Understanding
NC3: Reflecting
